# Prevalence and clinical associations of myositis antibodies in a large cohort of interstitial lung diseases

**DOI:** 10.1371/journal.pone.0277007

**Published:** 2022-11-03

**Authors:** Sofia A. Moll, Mark G. J. P. Platenburg, Anouk C. M. Platteel, Adriane D. M. Vorselaars, Montse Janssen Bonàs, Raisa Kraaijvanger, Claudia Roodenburg-Benschop, Bob Meek, Coline H. M. van Moorsel, Jan C. Grutters

**Affiliations:** 1 Department of Pulmonology, St. Antonius Hospital Nieuwegein, Centre for Interstitial Lung Diseases, Nieuwegein, The Netherlands; 2 Department of Medical Microbiology and Immunology, St. Antonius Hospital Nieuwegein, Nieuwegein, The Netherlands; 3 Division Heart & Lungs, University Medical Centre Utrecht, Utrecht, The Netherlands; Medizinische Hochschule Hannover, GERMANY

## Abstract

**Background:**

Serologic testing for autoantibodies is recommended in interstitial lung diseases (ILDs), as connective tissue diseases (CTDs) are an important secondary cause. Myositis antibodies are associated with CTD-ILD, but clinical associations with other ILDs are unclear. In this study, associations of myositis antibodies in various ILDs were evaluated.

**Methods:**

1463 ILD patients and 116 healthy subjects were screened for myositis antibodies with a line-blot assay on serum available at time of diagnosis. Additionally, bronchoalveolar lavage fluid (BALf) was analysed.

**Results:**

A total of 394 patients demonstrated reactivity to at least one antibody, including anti-Ro52 (36.0%), anti-Mi-2β (17.3%) and anti-Jo-1 (10.9%). Anti-Jo-1 (OR 6.4; p<0.100) and anti-Ro52 (OR 6.0; p<0.001) were associated with CTD-ILD. Interestingly, anti-Mi-2β was associated with idiopathic pulmonary fibrosis (IPF; OR 5.3; p = 0.001) and hypersensitivity pneumonitis (HP; OR 5.9; p<0.001). Furthermore, anti-Mi-2β was strongly associated with a histological usual interstitial pneumonia (UIP) pattern (OR 6.5; p < 0.001). Moreover, anti-Mi-2β reactivity was identified in BALf and correlated with serum anti-Mi-2β (r = 0.64; p = 0.002). No differences were found in survival rates between ILD patients with and without serum Mi-2β reactivity (hazard ratio 0.835; 95% CI 0.442–1.575; p = 0.577).

**Conclusion:**

In conclusion, novel associations of antibody Mi-2β with fibrotic ILD were found. Furthermore, serum anti-Mi-2β was associated with a histological UIP pattern and presence of anti-Mi-2β in BALf. Possibly, anti-Mi-2β could be implemented as a future diagnostic biomarker for fibrotic ILD.

## Introduction

Interstitial lung diseases (ILDs) are a group of heterogeneous, diffuse parenchymal lung diseases, characterized by inflammation and/or fibrosis of the pulmonary interstitium. ILDs can be idiopathic or secondary to known causes including environmental exposures, drugs or connective tissue disease (CTD) [1–4]. In 15% of patients with ILD an underlying CTD is identified [3, 5]. Distinguishing CTD related ILD (CTD-ILD) from other ILD is challenging as clinical, functional, radiological and pathological characteristics could be similar [6]. Moreover, an interstitial pneumonia (IP) may be the first or lone clinical manifestation of an associated CTD [4, 6]. In general, outcomes on treatment response to immunosuppressive therapy and survival are better in CTD-ILD compared to the majority of other ILDs [5–10]. Thus, discriminating these conditions in the diagnostic work-up is essential.

Serologic testing for autoantibodies by a myositis blot is recommended in pulmonary fibrosis suspected for an underlying CTD and includes myositis specific antibodies (MSA) and myositis associated antibodies (MAA) [1, 3, 4, 6, 11–16]. MSA and MAA are found in patients with idiopathic interstitial myopathies but also occur in patients with rheumatic diseases including CTD-ILDs [3–6, 11–23]. Presence of t-RNA synthase antibodies are strongly associated with ILD in antisynthetase syndrome (ASS), dermatomyositis (DM) and polymyositis (PM) [6, 20, 23–25]. Furthermore, a combination of t-RNA synthase antibodies and anti-Ro52/SSA is characterized by chronic and severe ILD [6, 19].

Myositis antibodies have also been identified in hypersensitivity pneumonitis (HP) and idiopathic IPs [11, 16, 26–28]. Although it is known that an IP can precede future CTD [3, 6], evidence is scarce on the clinical relevance of antibody positivity in patients meeting established criteria for other ILD. Therefore, it remains unclear how positive serologic testing in these ILDs should be interpret in clinical practice. Possibly, certain antibodies could be more associated with ILD features such as fibrosis than other characteristics of CTD.

To date, no major studies have compared myositis antibody positivity between CTD-ILD and other ILD. The aim of this study was to evaluate prevalence and clinical associations of myositis antibodies in these patients to enhance the diagnostic performance of serologic testing.

## Methods

### Patient selection

A retrospective cohort study was performed at the St Antonius ILD Centre of Excellence Nieuwegein, a tertiary ILD centre in the Netherlands. The majority of ILD patients were diagnosed between 2000 and 2019. The myositis blot used at our hospital was first introduced in 2012. All patients diagnosed with ILD before 2012, have not undergone a diagnostic myositis blot during their diagnostic work up. Subjects who had been tested for myositis antibodies during diagnostic work-up were evaluated. In addition, ILD patients who at first were not screened for myositis antibodies during their diagnostic work-up, were evaluated for the presence of antibodies in serum collected at date of diagnosis by a line-blot assay, run between 05–2019 and 07–2019. In addition, serum of healthy, non-ILD blood donors were screened for myositis antibodies as controls. For details on serum storage and sampling, see S1 File.

Diagnosis of ILD was assessed according to official ATS/ERS recommendations in a multidisciplinary discussion with an ILD pulmonologist, experienced thoracic radiologist, and a pathologist, when required [29]. All patients with pulmonary fibrosis were screened for an underlying CTD by the pulmonologist and referred to the rheumatologist for further diagnostic work-up if a CTD was suspected. During the multidisciplinary discussion, antibody reactivity was mainly interpreted by the rheumatologist. Furthermore, patients were referred to the rheumatologist during follow-up if an (ongoing) inflammation, suspected for autoimmune activity, was observed.

Patients were classified as having a CTD-ILD or ILD without established CTD (non-CTD-ILD). Patients were checked for any revisions of the ILD diagnosis during two years of follow-up, as an IP can precede an associated CTD often within two years after onset of disease [3, 6]. CTD-ILDs included antisynthetase syndrome (ASS), Sjogren’s syndrome, rheumatoid arthritis associated ILD (RA-ILD), systemic sclerosis (Ssc), dermatomyositis (DM), polymyositis (PM), immune mediated necrotizing myopathy (IMNM), inclusion body myositis (IBM), overlap myositis, systemic lupus erythematosus (SLE), mixed CTD and other CTD-ILD. Non-CTD-ILDs included idiopathic pulmonary fibrosis (IPF), hypersensitivity pneumonitis (HP), unclassifiable idiopathic interstitial pneumonia (Unclassifiable IIP), non-specific interstitial pneumonia (NSIP), cryptogenic organizing pneumonia (COP) and other ILD.

Patients with reactivity on a positive level against one or more antibodies were included for analysis. Only the first sample was included if more than one sample of a given patient was present. Subjects with solely weakly positive or negative antibody reactivity were excluded.

Baseline characteristics included pulmonary function tests, which were performed according to ATS/ERS recommendations [29]. Serum pneumoproteins including cancer antigen 15–3 (CA 15–3), CC chemokine ligand 18 (CCL18), Clara cell secretory protein (CC16), chitinase-3-like protein 1 (YKL-40) and surfactant protein D (SP-D) were evaluated as well. Characteristics on high-resolution computed tomography (HRCT) scans and in lung biopsies (when available) were classified as a pattern of usual interstitial pneumonia (UIP), probable UIP, indeterminate UIP or alternative diagnosis according to recent ATS/ERS recommendations [29]. Furthermore, prevalence of clinical symptoms was evaluated in patients with CTD-ILD. Autoimmune phenomena included for analysis were arthralgia, arthritis, Raynaud’s phenomenon, sicca complaints, mechanic’s hands, myalgia and muscle weakness.

The study was approved by the St Antonius institutional review under protocol number 842002003 and patients provided written informed consent for research purposes.

### Determination of antibodies

Antibodies were detected in serum using a line-blot assay (EUROLINE Autoimmune Inflammatory Myopathies, EUROIMMUN, Lübeck, Germany), in collaboration with Biognost. The assay identified MSA including antibodies against Jo-1, EJ, OJ, PL-7, PL-12, Mi-2α, Mi-2β, TIF1-γ, MDA5, NXP2 and SAE1 and MAA, including antibodies against Ku, PM/Scl-75, PM/Scl-100 and Ro52. Analysis of the immunoblot strips was performed with the EUROLINEScan software (EUROIMMUN, Lübeck, Germany) according to manufacturer’s recommendations as described for the EUROLINE Autoimmune Inflammatory Myopathies line blot assay. Strips were scored as negative, weakly positive and positive, which corresponds with intensity levels of respectively 0–10, 11–25 and >25. Antibody reactivity on a positive and weakly positive intensity level was separately evaluated.

### Statistical analysis

Baseline characteristics were expressed as numbers and percentages or mean and standard deviation. Continuous and categorical variables were tested with a student’s T-test/one-way ANOVA and Chi-Square test/Fisher’s Exact test respectively. A binary logistic regression analysis was performed in CTD-ILD and non-CTD-ILD for testing antibody level and association, expressed as general odds ratios. Survival plots were created using Kaplan Meier curves. Survival time was expressed in months from date of diagnosis. Survival was censored after death or lung transplantation. The statistical analysis was performed by software IBM SPSS version 24.0. A p-value less than 0.05 was considered as statistically significant.

## Results

### Baseline characteristics

A total of 1463 ILD patients were screened for myositis antibodies. One hundred CTD-ILD and 294 non-CTD-ILD patients were positive for at least one antibody and were included in this study. Baseline characteristics are listed in Table 1 and per ILD diagnosis in the supplementary data (S1 and S2 Tables in S1 File). Of CTD-ILD patients, a revision on the initial diagnosis (all formerly diagnosed as NCIP) was made in 15 (15%) patients. Mean duration between initial diagnosis and revision was 12.3 months (SD 8.8; range 2–23 months).

**Table 1:** Baseline characteristics of 394 patients with ILD.

Subjects	All	CTD-ILD	Non-CTD-ILD	P^e^
N	394	100	294	
Age	63.6 (11.2)	59.1 (11.5)	65.1 (10.7)	< 0.001
Sex (m), %	242 (61.4)	46 (46.0)	196 (66.7)	< 0.001
History of smoking, %	272 (68.7)	56 (56.0)	212 (72.4)	0.007
**Pulmonary function test** ^**a**^				
FVC	81.0 (20.1)	80.4 (23.2)	81.2 (19.0)	0.751
FEV1	83.3 (37.9)	86.3 (67.9)	82.3 (19.8)	0.571
DLCO	46.9 (16.2)	49.3 (15.0)	46.2 (16.5)	0.101
**Pneumoprotein** ^**b**^				
CA 15–3	64.9 (64.2)	51.2 (46.8)	69.8 (68.9)	0.003
CC16	20.2 (26.1)	17.2 (46.8)	21.2 (15.1)	0.410
CCL18	174.2 (112.7)	164.7 (93.2)	177.3 (118.3)	0.331
SP-D	113.4 (194.1)	108.7 (175.1)	115.1 (200.8)	0.768
YKL-40	148.4 (133.7)	140.8 (122.0)	151.2 (137.8)	0.724
**HRCT scan** ^**c**^				
UIP	96 (24.9)	11 (11.3)	85 (29.4)	< 0.001
Probable UIP	47 (12.2)	7 (7.2)	40 (13.8)	0.084
Indeterminate	75 (19.4)	24 (24.7)	51 (17.6)	0.126
Alternative	168 (43.5)	55 (56.7)	113 (39.1)	0.002
**Histopathology** ^**d**^				
UIP	24 (20.5)	-	24 (27.0)	0.002
Probable UIP	12 (10.3)	5 (17.9)	7 (7.9)	0.129
Indeterminate	20 (17.1)	5 (17.9)	15 (16.9)	0.902
Alternative	61 (52.1)	18 (64.3)	43 (48.3)	0.140

Data are expressed as mean and standard deviation or numbers and percentage within the diagnosis group.

CTD = connective tissue disease; ILD = interstitial lung disease; non-CTD-ILD = ILD without established CTD; FVC = forced vital capacity; FEV1 = forced expiratory volume in 1 second; DLCO = diffusing capacity of the lung for carbon monoxide; CA 15–3 = cancer antigen 15–3; CCL18 = CC chemokine ligand 18; CC16 = Clara cell secretory protein; SP-D = surfactant protein D; YKL-40 = chitinase-3-like protein 1; UIP = usual interstitial pneumonia

^a^ n = 383, data expressed mean and standard deviation in percentage of predicted

^**b**^ n = 383, data expressed as mean and standard deviation in kU/l (CA 15–3) or ng/ml (CC16, CCL18, SP-D, YKL-40)

^**c**^ n = 386, data expressed as numbers and percentage

^**d**^ n = 117, data expressed as numbers and percentage

^**e**^ p < 0.05, differences between CTD-ILD and non-CTD-ILD are calculated by a two-side unpaired T-test/One way Anova for continuous variables or Chi-Square test/Fisher’s Exact test for dichotomous variables.

Significant differences between CTD-ILD and non-CTD-ILD were found for age (p<0.001), sex (p<0.001), smoking history (p = 0.007) and serum CA 15–3 (p = 0.003). Furthermore, radiological (p<0.001) and histopathological (p = 0.002) UIP patterns were predominantly observed in non-CTD-ILDs (range 4.2–82.4%) including IPF.

In CTD-ILD patients, presence of one or more autoimmune phenomena were common: arthralgia 75% (n = 75), arthritis 27% (n = 27), Raynaud’s phenomenon 44% (n = 44), sicca complaints 51% (n = 51), mechanic’s hands 11% (n = 11), myalgia 55% (n = 55) and muscle weakness 36% (n = 36). Prevalence of autoimmune phenomena per subtype CTD-ILD are listed in S1 Table in S1 File. Between groups, prevalence of arthritis was significantly different (p<0.001), with highest prevalence in patients with RA-ILD (n = 11; 100%).

Of CTD-patients, 86 patients (86%) showed non-UIP radiological patterns, i.e. patterns of probable UIP, indeterminate UIP or alternative diagnosis. In detail, radiological patterns of NSIP (n = 47), OP (n = 23), ground glass nodules (n = 4), lymphocytic interstitial pneumonitis (LIP; n = 3) and other radiological dominant patterns, including cysts (n = 1) and nodules (n = 1) were described. For non-CTD-ILD patients, non-UIP radiological patterns were seen in 69.4% of patients (n = 204). In detail, radiological patterns of NSIP (67.2%), OP (11.8%), ground glass nodules (11.3%), LIP (1.0%), emphysema (3.4%), cysts (1.5%) and nodules (3.9%) were observed.

### Frequencies of myositis antibodies in patients with ILD

Antibody prevalence was evaluated for all ILD, 116 healthy controls (Table 2) and per ILD diagnosis (S3 and S4 Tables in S1 File). On a positive intensity level, the most prevalent antibodies in ILD were Ro52 (36.0%) and Mi-2β (17.3%), followed by Jo-1 (10.9%) and SRP (7.4%, Table 2). Antibody prevalence was significantly higher in ILD compared to controls except for antibodies EJ, MDA5, Mi-2α, NXP2 and OJ. Anti-Ro52 reactivity was high in ASS (83.9%) and Sjogren’s syndrome (92.3%, S3 Table in S1 File) but also observed in unclassifiable IIP, NSIP and COP (range 27.3–54.8%; S4 Table in S1 File). Prevalence of antibodies Mi-2β, SRP and Ku was significantly higher in non-CTD-ILD compared to CTD-ILD (all p<0.05, see Table 2), in particular in IPF (respectively 26.5%, 11.8% and 8.8%) and unclassifiable IIP (respectively 17.9%, 14.2% and 7.5%; S4 Table in S1 File). Mi-2β antibodies were observed in HP as well (26.4%).

**Table 2:** Frequency of myositis antibodies in ILD patients and healthy controls.

Antibody	All ILD	CTD-ILD	Non-CTD-ILD	P^a^	Healthy controls	P^b^
N	394	100	294		116	
**MSA**						
EJ	10 (2.5)	5 (5.0)	5 (1.7)	0.070	1 (0.9)	0.470
Jo-1	43 (10.9)	27 (27.0)	16 (5.4)	<0.001	-	<0.001
MDA5	8 (2.0)	2 (2.0)	6 (2.0)	0.980	-	0.208
Mi-2α	6 (1.5)	1 (1.0)	5 (1.7)	0.621	-	0.345
Mi-2β	68 (17.3)	7 (7.0)	61 (20.7)	0.002	1 (0.9)	<0.001
NXP2	7 (1.8)	4 (4.0)	3 (1.0)	0.051	-	0.359
OJ	7 (1.8)	1 (1.0)	6 (2.0)	0.496	-	0.359
PL-12	21 (5.3)	11 (11.0)	10 (3.4)	0.003	-	0.006
PL-7	24 (6.1)	5 (5.0)	19 (6.5)	0.597	-	0.006
SAE1	14 (3.6)	2 (2.0)	14 (4.8)	0.026	-	0.047
SRP	29 (7.4)	2 (2.0)	27 (9.2)	0.017	-	0.002
TIF1-γ	24 (6.1)	1 (1.0)	23 (7.8)	0.014	-	0.006
**MAA**						
Ku	20 (5.1)	1 (1.0)	19 (5.4)	0.032	-	0.011
PM/Scl 100	22 (8.4)	13 (13.0)	20 (6.5)	0.053	1 (0.9)	0.013
PM/Scl 75	46 (11.7)	13 (13.0)	33 (11.2)	0.633	-	<0.001
Ro52	142 (36.0)	67 (67.0)	75 (25.5)	<0.001	2 (1.7)	<0.001

Data are expressed as numbers and percentage of positive antibodies within each ILD diagnosis group. Weakly positive antibodies are excluded.

CTD = connective tissue disease; ILD = interstitial lung disease; non-CTD-ILD = ILD without established CTD

^a^ p < 0.05, difference between CTD-ILD and other ILD patients calculated by Chi-Square test or Fisher’s Exact test.

^b^ p < 0.05, difference between all ILD and healthy controls calculated by Chi-Square test or Fisher’s Exact test

### Associations between antibody level and ILD

Associations between antibody reactivity and ILD were evaluated (Table 3). At the positive intensity level, strong associations were found of antibodies Jo-1 (OR 6.4; p<0.001), PL-12 (OR 3.4; p = 0.007) and Ro52 (OR 6.0; p<0.001) with CTD-ILD. Furthermore, odds ratios of less than one were found with antibodies Mi-2β (OR 0.3; p = 0.002), SRP (OR 0.2; p = 0.026) and Ku (OR 0.1; near-significant; p = 0.058) with CTD-ILD. Expressed as a reversed odds ratio (1/OR), corresponding ratios were 2.7, 5.3 and 7.1 respectively, indicating odds in favour of non-CTD-ILD. A sub analysis of CTD-ILD compared to IPF showed strong associations of antibodies Mi-2β (OR 0.2; 1/OR 5.3; p = 0.001), Ku (OR 0.1; 1/OR 10; p = 0.034) and SRP (OR 0.1; 1/OR 7.7; p = 0.013; S5 Table in S1 File) with IPF. Similarly, antibodies Mi-2β (OR 3.2; p = 0.015), Ku (OR 5.0 p = 0.046) and TIF1-γ (OR 16.7; p = 0.008) were associated with unclassifiable IIP compared to CTD-ILD. Mi-2β antibody was associated with HP compared to CTD-ILD as well (OR 5.9; p<0.001).

**Table 3:** Associations of myositis antibodies with ILD patients.

Antibody	CTD-ILD (n = 100)			Non-CTD-ILD (n = 294)					
	Number Neg	Number Weak pos	Number Pos	Number Neg	Number Weak pos	Number Pos	OR p	95% CI^b^	p^c^
							OR wp^a^		
EJ	95	-	5	289	-	5	3.0	0.86–10.76	0.084
							-		
Jo-1	72	1	27	271	7	16	6.4	3.25–12.42	<0.001
							0.5	0.07–4.44	0.565
Ku	98	1	1	262	13	19	0.1	0.02–1.07	0.058
							0.2	0.03–1.59	0.130
MDA5	97	1	2	277	11	6	1.0	0.19–4.80	0.952
							0.3	0.03–2.04	0.199
Mi-2α	99	-	1	285	4	5	0.6	0.07–4.99	0.616
							-		
Mi-2β	91	2	7	211	22	61	0.3	0.12–0.60	0.002
							0.2	0.05–0.92	0.038
NXP2	95	1	4	287	4	3	4.0	0.89–18.3	0.071
							0.8	0.08–6.84	0.803
OJ	99	-	1	284	4	6	0.5	0.06–4.02	0.497
							-		
PL-12	89	-	11	278	6	10	3.4	1.41–8.36	0.007
							-		
PL-7	95	-	5	270	5	19	0.8	0.27–2.06	0.574
							-		
PM/Scl 100	87	-	13	260	14	20	1.9	0.93–4.07	0.078
							-		
PM/Scl 75	86	1	13	249	12	33	1.1	0.57–2.27	0.707
							0.2	0.03–1.88	0.175
Ro52	31	2	67	208	11	75	6.0	3.63–9.89	<0.001
							1.2	0.258–5.77	0.802
SAE1	98	2	-	271	9	14	-		
							0.6	0.13–2.89	0.538
SRP	90	8	2	232	35	27	0.2	0.04–0.82	0.026
							0.6	0.26–1.14	0.198
TIF1-γ	96	3	1	265	6	23	0.1	0.02–0.90	0.039
							1.4	0.34–5.63	0.653

CTD = connective tissue disease; ILD = interstitial lung disease; non-CTD-ILD = ILD without established CTD

^**a**^ OR: odds ratio for positive level (OR p); odds ratio for weak positive level (OR wp).

^b^ 95% confidence interval of odds ratio’s

^c^ Logistic regression analysis of CTD versus other patients with positive, weak positive and negative antibody, with predicted probability for CTD-ILD.

### Associations between antibody level and radiological and histological characteristics

Next, associations of antibodies with radiological and histological patterns were evaluated. Anti-SAE1 was associated with a radiological UIP pattern (OR 3.2; p = 0.036). Anti-Ro52 was associated with both radiological (OR 2.7; p<0.001) and histological non-UIP patterns (OR 0.16; 1/OR 6.3; p = 0.005; see Table 4). Interestingly, antibody Mi-2β was strongly associated with a histological UIP pattern (OR 6.5; p<0.001) but not significantly associated with a radiological UIP pattern. Concerning non-UIP radiological patterns (n = 47) in serum Mi-2β positive ILD patients, patterns of NSIP (83.8%), OP (12.8) and ground glass nodules (10.6%) were most prevalent. However, evaluation of ILD patients classified per radiological pattern (from UIP to alternative diagnosis) showed that the association of anti-Mi-2β with histological UIP persisted within each radiological group (OR range 4.3–10, all p<0.05), indicating that the association of serum Mi-2β antibodies with histological UIP was independent of the patients’ corresponding radiological pattern. Other antibodies were not significantly associated with radiological patterns.

**Table 4:** Associations of myositis antibodies with a histological UIP pattern and non-UIP pattern.

Antibody	UIP (n = 24)			Non-UIP (n = 93)					
	Number Neg	Number Weak pos	Number Pos	Number Neg	Number Weak pos	Number Pos	OR p	95% CI^b^	p^c^
							OR wp^a^		
EJ	24	-	-	92	-	1	-		
							-		
Jo-1	21	1	2	83	1	9	0.88	0.18–4.37	0.874
							3.95	0.24–65.84	0.338
Ku	23	1	-	91	1	1	-		
							3.96	0.24–65.67	0.337
MDA5	23	1	-	91	1	1	-		
							3.96	0.24–65.67	0.337
Mi-2α	22	1	1	88	3	2	1.33	0.13–13.45	0.807
							2.00	0.17–23.07	0.579
Mi-2β	10	3	11	77	3	13	6.52	2.31–18.41	<0.001
							7.70	1.36–43.46	0.021
NXP2	24	-	-	92	-	1	-		
							-		
OJ	23	1	-	92	-	1	-		
							-		
PL-12	22	2	-	81	1	11	-		
							7.36	0.64–85.01	0.110
PL-7	21	1	2	86	2	5	1.64	0.30–9.04	0.571
							2.05	0.18–23.67	0.566
PM/Scl 100	22	-	2	78	3	12	0.59	0.12–2.84	0.511
							-		
PM/Scl 75	18	2	4	76	2	15	1.13	0.33–3.80	0.848
							4.22	0.56–32.03	0.164
Ro52	20	1	3	45	6	42	0.16	0.04–0.58	0.005
							0.38	0.04–3.32	0.378
SAE1	22	-	2	90	1	2	4.09	0.55–30.67	0.171
							-		
SRP	15	7	2	78	11	4	2.60	0.44–15.50	0.294
							3.31	1.11–9.91	0.033
TIF1-γ	19	3	2	87	1	5	1.83	0.33–10.16	0.489
							13.74	1.35–139.36	0.027

UIP = usual interstitial pneumonia

^**a**^ OR: odds ratio for positive level (OR p); odds ratio for weakly positive level (OR wp).

^b^ 95% confidence interval of odds ratio’s

^c^ Logistic regression analysis of histological UIP versus non UIP patients with positive, weakly positive and negative antibody, with predicted probability for UIP.

### Survival analysis

A survival analysis was performed to explore the prognostic value of antibody reactivity against Mi-2β in ILD patients. Overall, mean follow-up was 66.7 months (SD 50.5). Median survival of CTD-ILD patients (47 months) was significantly higher compared to non-CTD-ILD patients (median 33 months; p = 0.001). ILD patients with reactivity against Mi-2β showed no difference in survival (median 31.0 months) compared to patients without reactivity against Mi-2β (median 29.1 months; hazard ratio 0.835; 95% CI 0.442–1.575; p = 0.577). Within the CTD-ILD group, no difference was observed in survival between patients with and without reactivity against Mi-2β (p = 0.993). For the non-CTD-ILD group, no significant difference between patients with and without reactivity against Mi-2β was observed as well (p = 0.352).

### Antibody expression in bronchoalveolar lavage fluid (BALf)

An analysis was performed in BALf with regard to the association of antibody Mi-2β with histological UIP pattern. First, BALfs were retrieved of patients with serum Mi-2β antibodies on a positive intensity level and of a subset of patients without Mi-2β antibodies. BALf was tested for the presence of anti-Mi-2β by the line-blot assay (see S1 File). To determine the extent of possible leakage of blood plasma products to the alveoli, an albumin BALf/serum ratio was calculated and used as an indicator for antibody leakage. Nine serum Mi-2β positive ILD and eleven serum Mi-2β negative ILD with available BALfs were included (Table 5). Of serum Mi-2β positive ILD, one patient (IPF) demonstrated Mi-2β reactivity on a positive level in BALf, one patient (HP) on a weakly positive level and three (n = 2 HP and n = 1; RA-ILD) on a borderline weakly positive level (intensity level 6–10). Serum Mi-2β negative ILD patients did not show any Mi-2β reactivity in BALf. No differences were found in albumin BALf/serum ratios between serum Mi-2β positive and negative ILD patients (p = 0.849). In addition, albumin BALf/serum ratios in serum Mi-2β positive ILD were not different as well between subjects with and without Mi-2β reactivity in BALf (p = 0.568). Mi-2β reactivity on a combined (borderline) weakly positive and positive level in BALf was strongly associated with concurrent serum Mi-2β reactivity (r = 0.64; p = 0.002). Mi-2β reactivity on a positive level only in BALf was not significantly associated with serum Mi-2β reactivity.

**Table 5:** Mi-2β measurement in bronchoalveolar lavage fluid in ILD patients.

Patient	Diagnosis	Age (y)	Sex	HRCT scan	Histopathology	Serum Mi-2β	BALf Mi-2β	Albumin BALf/serum^a^
1	IPF	47	M	Probable UIP	-	Pos	Pos	1.29
2	HP	75	M	Alternative	-	Pos	Weak pos	1.27
3	HP	47	M	Alternative	Alternative	Pos	Borderline	0.22
4	HP	73	M	Alternative	-	Pos	Borderline	4.09
5	RA-ILD	54	M	Alternative	-	Pos	Borderline	1.67
6	HP	59	M	Alternative	-	Pos	Neg	1.49
7	HP	73	F	UIP	-	Pos	Neg	0.99
8	Unclassifiable IIP	72	F	Probable UIP	-	Pos	Neg	1.25
9	Unclassifiable IIP	50	F	UIP	Indeterminate UIP	Pos	Neg	1.29
10	IPF	74	M	UIP	-	Neg	Neg	1.44
11	COP	73	F	Alternative	Alternative	Neg	Neg	1.65
12	Sjogren’s syndrome	75	M	Alternative	-	Neg	Neg	1.14
13	IPF	80	M	UIP	-	Neg	Neg	0.24
14	Sjogren’s syndrome	73	F	Probable UIP	-	Neg	Neg	1.70
15	IPF	65	M	Probable UIP	UIP	Neg	Neg	1.07
16	IPF	76	M	UIP	-	Neg	Neg	0.87
17	ASS	62	M	Indeterminate UIP	Probable UIP	Neg	Neg	1.56
18	ASS	47	M	Alternative	-	Neg	Neg	4.05
19	Unclassifiable IIP	80	M	Alternative	-	Neg	Neg	0.56
20	HP	72	M	UIP	-	Neg	Neg	4.21

ILD = interstitial lung disease; IPF = idiopathic pulmonary fibrosis; HP = hypersensitivity pneumonitis; RA-ILD; rheumatoid arthritis associated interstitial lung disease; Unclassifiable IIP = unclassifiable idiopathic interstitial pneumonia; COP = cryptogenic organizing pneumonia; ASS: antisynthetase syndrome; HRCT = high resolution computed tomography; UIP = usual interstitial pneumonia; BALf = bronchoalveolar lavage fluid

Pos = antibody reactivity on a positive intensity level; Weak pos = antibody reactivity on a weakly positive intensity level; Borderline = antibody reactivity on a borderline weakly positive intensity level (6–10); neg = no antibody reactivity.

^a^ ratio of albumin level in BALf (mg/l) and serum (g/l).

## Discussion

In this study, we evaluated prevalence, clinical characteristics and associations of myositis antibodies in a large cohort of ILD. Antibodies Jo-1 and Ro52 were strongly associated with CTD-ILD. Strikingly, we demonstrated stronger associations of anti-Mi-2β positivity with IPF, HP and unclassifiable IIP compared to CTD-ILD. Furthermore, Mi-2β antibody was strongly associated with a histological pattern of UIP. Interestingly, anti-Mi-2β reactivity was detected in BALf and correlated with serum Mi-2β reactivity in ILD. No differences were found in survival rates between ILD patients with and without serum Mi-2β reactivity. To date, the clinical value of positive myositis antibodies in other ILDs including idiopathic IP remains unclear. Possibly, testing of autoantibody Mi-2β in particular could be used as a diagnostic biomarker for fibrotic ILD in clinical practice.

Antibodies against t-RNA synthetases and Ro52 were demonstrated in various ILDs. Antibody Jo-1 is considered as a predictor for ILD and was observed in 27% of CTD-ILD, compared to 30–50% found in DM with ILD [6, 20]. Prevalence of MSA was high in non-CTD-ILD compared to studies with idiopathic IP, in which 6.6–24% of the subjects showed MSA including Jo-1, EJ, NXP-2, PL-7, TIF1-γ and SRP [11, 16, 26]. Contrary, antibody PL-12 was infrequently observed in our IPF cohort (1.5%) compared to an IPF study (5.3%) [27]. Furthermore, patients with idiopathic IP and positive for antibodies EJ, PL-7 or PL-12 were radiologically and/or histologically characterized by a pattern of NSIP or UIP [16, 11, 28], which is in agreement with characteristics of IPF and unclassifiable IIP in our cohort. Antibody frequencies of PL-12 and PL-7 in ASS (both 9.7% respectively) were low compared to ASS-ILD studies (range 60–77%) [23, 24, 26]. However, similarities in histological characteristics were observed in anti-PL-12 positive ILD (non-UIP) compared to findings in anti-PL-12 positive ASS (NSIP) [25]. Furthermore, Ro52 antibodies were frequently observed in both our CTD-ILDs (67%) and in a study with CTD-ILDs including PM/DM and Sjogren’s syndrome (60%) [30]. Prevalence of antibody Ro52 in IPF (14.7%) was in congruence with previous IPF research (15.8%) [28].

Novel findings on associations of antibody Mi-2β with fibrotic ILDs were found, in particular IPF. In DM research, Mi-2β antibodies were demonstrated in 4–14% of the subjects and correlated with the presence of an IP [15, 20]. However, Mi-2β antibodies have not been described in patients with IPF, HP or unclassifiable IIP. Our study adds to previous research that serum Mi-2β antibodies are associated with ILDs without established CTD, including idiopathic IPs. In addition, we are the first to demonstrate anti-Mi-2β reactivity in BALf of ILD patients and its strong association with concurrent serum Mi-2β reactivity. It is known that the Mi-2β antigen is part of the NuRD complex, which is regulated by the chromatin remodelling complex gene CDH4 [31]. CDH4 is essential for specification of early B-cell lineage transcriptional program in lymphocytes [32]. Inactivation of the CDH4/NuRD complex in mural models lead to extensive cardiac fibrosis due to de-differentiation of cardiac myocytes [33]. In addition, high expression of Mi-2β was demonstrated in regenerating myofibers in mice. Moreover, Mi-2β expression was higher in muscle biopsies of DM patients, of which one had concurrent serum Mi-2β antibodies as well, compared to healthy controls [34, 35]. It could be hypothesized that the antibodies against Mi-2β are formed during the remodelling process following destruction of cells important for the structural integrity of alveoli, particularly alveolar type II pneumocytes, and release of the NuRD complex. This loss in structural integrity will be compensated by induction of fibrosis. Possibly, antibody Mi-2β could be used as a distinctive and diagnostic biomarker for pulmonary fibrosis with absence of extra thoracic features.

This study was performed with patients whom were all diagnosed by a standardized multidisciplinary discussion in a tertiary ILD centre in the Netherlands. It is the first study to describe prevalence and associations of myositis antibodies in a large cohort of patients with other ILD compared with CTD-ILD and healthy subjects. This retrospective study has some limitations, as selection bias of more severely impaired patients with pulmonary fibrosis is possible in a referral centre. Although pulmonary involvement in CTD often arises within the first two years after onset of disease [6], it might be possible that a diagnosis bias could have occurred, using a two-year cut-off for checking on revisions on the initial ILD diagnosis. It might be possible that, despite multidisciplinary discussion including a rheumatologist, clinical signs suggestive of an underlying CTD have been unnoticed or developed after two years of onset of ILD. Therefore, clinicians should stay aware for signs suggestive for an underlying CTD in ILD patients during follow-up. Furthermore, overall prevalence of myositis antibodies in both CTD-ILD and other ILD might be overestimated, as only patients with at least one positive myositis antibody were included for analysis.

The findings of this research raise question why autoantibodies are present in idiopathic IP, including IPF. It is acknowledged that IPF is the result of chronic activated fibroblast-myofibroblasts after repetitive damage, leading to tissue remodelling and injury of alveolar type II pneumocytes [36, 37]. The role of inflammation is controversial though, and sometimes described as an epiphenomenon or even co-driver of disease [38]. Increased numbers of auto-antibody producing plasma cells in human fibrotic lung tissue have been described in multiple studies, summarized in [38]. Production of autoantibodies comes along with the increased expression of immunity- and inflammation-related genes [39], including many B cell related genes [39, 40] and tertiary lymph nodes [38, 41] during development of fibrosis. This supports a model in which autoreactive B cells are continuously primed and allowed to differentiate into plasma cells secreting autoantibodies. These may include long-lived autoreactive plasma cells as well [38], with the ability to survive in the bone narrow and continuously secrete autoantibodies in absence of antigen stimuli. Furthermore, these plasma cells are resistant to immunosuppressive or B-cell depleting therapy [42]. Thus, it could be hypothesized that certain autoantibodies, such as Mi-2β, are continuously produced in idiopathic IP and do not diminish after treatment with immunosuppressive therapy, whereas antibodies in active CTD-ILD would be more likely the result of short-lived autoreactive plasma cells which are sensitive for immunosuppressants. To investigate this hypothesis, a prospective study in CTD-ILD and non-CTD-ILD with multiple testing of Mi-2β antibodies before start and during therapy is imperative. Furthermore, a prospective cohort study is needed to confirm whether antibody positive ILD patients do not develop future auto immune features of an underlying CTD, as it is known that an IP can precede two years before clinical manifestations of an associated CTD [3, 6].

For future research, it would be interesting to investigate associations with antibody reactivity and therapeutic strategies, including anti-inflammatory drugs, used in patients with CTD-ILD and other ILD. Moreover, it would be interesting to assess whether anti-fibrotic therapy reduces anti-Mi-2β signal during follow-up in ILD. In clinical practice, a novel approach of testing and interpretation of autoantibodies could be implemented. In Ssc related ILD, which is regularly treated by immunosuppressive therapy, the anti-fibrotic drug nintedanib slowed the progression of ILD [43]. Conversely, IPF patients with circulating autoantibodies could possibly be approached as a phenotype, which might be sensitive for the combination of both anti-fibrotic and immunosuppressive drugs.

To date, the proposed classification of IPAF is a research concept and has not been implemented as an official ILD diagnosis yet [44]. It would be interesting for future search to investigate whether non-CTD-ILD patients who have not previously been tested for MSA/MAA and showing reactivity, would have meet the criteria for IPAF in retrospect.

In conclusion, we demonstrated associations of myositis antibodies including anti-Mi-2β in a large cohort of other ILDs compared to CTD-ILD. Possibly, Mi-2β antibody could be used as a diagnostic biomarker for fibrotic ILD in clinical practice.

## Supporting information

S1 File (DOCX)

## Abbreviations

ANA: Antinuclear antibody
ASS: Anti-synthetase syndrome
ATS: American Thoracic Society
BALf: Bronchoalveolar lavage fluid
CA 15–3: Cancer antigen 15–3
CC16: Clara cell secretory protein
CCL18: CC chemokine ligand 18
COP: Cryptogenic organizing pneumonia
CPFE: Combined pulmonary fibrosis and emphysema
CTD: Connective tissue disease
DIP: Desquamative interstitial pneumonia
DM: Dermatomyositis
Dlco: Diffusing capacity of the lung for carbon monoxide
ERS: European Respiratory Society
FVC: Forced vital capacity
FEV1: Forced expiratory volume in 1 second
HP: Hypersensitivity pneumonitis
HRCT: High-resolution computed tomography
IBM: Inclusion body myositis
IIP: Idiopathic interstitial pneumonia
ILD: Interstitial lung disease
IMNM: Immune mediated necrotizing myopathy
IP: Interstitial pneumonia
IPAF: Interstitial pneumonia with autoimmune features
IPF: Idiopathic pulmonary fibrosis
MAA: Myositis associated antibody
MSA: Myositis specific antibody
NSIP: Non-specific interstitial pneumonia
PFT: Pulmonary function test
PM: Polymyositis
RA: Rheumatoid arthritis
SLE: Systemic lupus erythematosus
SP-D: Surfactant protein D
Ssc: Systemic sclerosis
UIP: Usual interstitial pneumonia
YKL-40: Chitinase-3-like protein 1
