# Supplementary material for: Prevalence and clinical associations of myositis antibodies in a large cohort of interstitial lung diseases
Source: PLoS One. 2022 Nov 3;17(11):e0277007. doi: 10.1371/journal.pone.0277007 (PMC9632801; doi:10.1371/journal.pone.0277007)
Supplement: S1 File — (DOCX) [file pone.0277007.s001.docx]

**Supplement**

Storing and sampling of serum

At the St Antonius ILD Centre of Excellence, serum samples of ILD patients have been systematically stored in an ILD biobank for approximately 15 years for research purposes. Stored sera, sampled at time of diagnosis, was used to for this blot. Whole blood samples were centrifuged within 3 hours of collection, the serum was aliquoted in cryovials and stored at -80°C. One hour before analysis, one aliquot of the samples were thawed at room temperature. The samples used had not been previously thawed to avoid multiple freeze-thaw cycles.

Determination of antibodies in bronchoalveolar lavage fluid

Bronchoalveolar lavage fluid (BALf) was screened for Mi-2β antibodies by a line-blot assay on a immunoblot work station (EUROLINE Autoimmune Inflammatory Myopathies/EUROBlotMaster, EUROIMMUN, Lübeck, Germany), in collaboration with Biognost. Analysis of the immunoblot strips was performed with the EUROLINEScan software (EUROIMMUN, Lübeck, Germany) according to manufacturer’s recommendations as described for the EUROLINE Autoimmune Inflammatory Myopathies line blot assay. All patients underwent bronchoalveolar lavages, prior to the date of diagnosis, for diagnostic purposes. Samples were stored at -80 ̊C until analysis.

After the start of run of the EUROBlotMaster, 1.5 ml of sample buffer was automatically pipetted on the strips, followed by an incubation time of 5 minutes. Next, 0.75 ml of the sample buffer was extracted and replaced by 0.75 ml supernatant BALf (dilution 1:1).

In addition, albumin levels in BALf (mg/l) and corresponding baseline serum (g/l) were retrieved. Next, albumin BALf/serum ratio was calculated as a marker for albumin leakage from the serum to the intrapulmonary space. This ratio was used as an indicator of leakage of other blood plasma products, including myositis antibodies, from the serum to the alveoli.

| **Supplementary table 1** | | **Baseline characteristics of 100 CTD-ILD patients** | | | | | | | |  |  |
| --- | --- | --- | --- | --- | --- | --- | --- | --- | --- | --- | --- |
| **Subjects** | **All CTD-ILD** | **ASS** | **Sjogren’s syndrome** | **RA-ILD** | **Ssc** | **PM/DM** | **IBM** | **SLE** | **Mixed CTD-ILD** | **Other CTD-ILD** ^a^ | **P^f^** |
|  |  |  |  |  |  |  |  |  |  |  |  |
| N | 100 | 31 | 26 | 11 | 10 | 6 | 1 | 1 | 10 | 4 |  |
| Age | 59.1 (11.5) | 58.7 (10.2) | 57.2 (14.9) | 63.0 (5.8) | 59.5 (10.9) | 59.0 (7.7) | 69.0 (-) | 68.0 (-) | 57.5 (12.7) | 62.3 (16.1) | 0.879 |
| Sex (m), % | 46 (46.0) | 14 (45.2) | 5 (19.2) | 8 (72.7) | 7 (70.0) | 4 (66.7) | 1 (100.0) | 1 (100.0) | 3 (30.0) | 3 (75.0) | 0.009 |
| History of smoking, % | 56 (56.0) | 17 (58.6) | 12 (46.2) | 9 (81.8) | 6 (60.0) | 3 (50.0) | 1 (100.0) | 1 (100.0) | 6 (60.0) | 1 (25.0) | 0.401 |
| **Pulmonary function test^b^** |  |  |  |  |  |  |  |  |  |  |  |
| FVC | 80.4 (23.2) | 77.2 (22.1) | 81.3 (24.6) | 83.2 (23.4) | 80.5 (17.4) | 61.6 (27.0) | 96.7 (-) | 99.0 (-) | 87.6 (27.3) | 88.3 (23.9) | 0.602 |
| FEV1 | 86.3 (67.9) | 77.2 (20.7) | 80.4 (24.2) | 141.8 (189.7) | 83.8 (20.9) | 61.4 (20.9) | 97.6 (-) | 81.0 (-) | 83.1 (20.9) | 81.1 (23.5) | 0.361 |
| DLCO | 49.3 (15.0) | 44.9 (14.5) | 50.0 (13.7) | 46.9 (8.8) | 46.8 (14.4) | 52.9 (24.2) | 67.5 (-) | 56.0 (-) | 57.3 (16.3) | 56.3 (22.9) | 0.378 |
| **Pneumoprotein^c^** | |  |  |  |  |  |  |  |  |  |  |
| CA 15-3 | 51.2 (46.8) | 47.1 (40.3) | 61.9 (60.5) | 48.5 (35.3) | 51.6 (32.9) | 57.0 (30.2) | 23.0 (-) | 34.0 (-) | 48.4 (66.7) | 29.0 (29.7) | 0.932 |
| CC16 | 17.2 (46.8) | 12.7 (7.6) | 14.3 (7.9) | 54.1 (129.5) | 8.7 (3.4) | 15.0 (8.6) | 13.0 (-) | 19.0 (-) | 12.8 (10.0) | 8.3 (1.7) | 0.371 |
| CCL18 | 164.7 (93.2) | 180.9 (97.5) | 141.2 (101.0) | 175.5 (76.6) | 212.5 (87.7) | 132.2 (80.2) | - | 217.0 (-) | 157.9 (98.5) | 108.5 (57.5) | 0.459 |
| SP-D | 108.7 (175.1) | 79.5 (110.3) | 158.0 (251.2) | 118.5 (134.5) | 143.1 (241.8) | 45.4 (46.7) | 276.0 (-) | 19.5 (-) | 89.4 (125.0) | 14.2 (12.6) | 0.599 |
| YKL-40 | 140.8 (122.0) | 140.9 (113.4) | 125.5 (107.6) | 151.6 (79.2) | 165.3 (137.4) | 177.1 (251.7) | 45.0 (-) | 261.9 (-) | 125.1 (145.0) | 122.8 (68.3) | 0.924 |
| **HRCT scan^d^** |  |  |  |  |  |  |  |  |  |  |  |
| UIP | 11 (11.3) | 3 (10.0) | 1 (3.8) | 5 (45.5) | 1 (11.1) | 1 (16.7) | - | - | - | - | 0.088 |
| Probable UIP | 7 (7.2) | - | 4 (15.4) | 2 (18.2) | 1 (11.1) | - | - | - | - | - | 0.188 |
| Indeterminate | 24 (24.7) | 8 (26.7) | 8 (30.8) | - | 2 (22.2) | - | 1 (100.0) | 1 (100.0) | 3 (33.3) | 1 (25.0) | 0.040 |
| Alternative | 55 (56.7) | 19 (63.3) | 13 (50.0) | 4 (36.4) | 5 (55.6) | 5 (83.3) | - | - | 6 (66.7) | 3 (75.0) | 0.334 |
| **Histopathology^e^** | |  |  |  |  |  |  |  |  |  |  |
| UIP | - | - | - | - | - | - | - | - | - | - | - |
| Probable UIP | 5 (17.9) | 2 (33.3) | 2 (16.7) | - | - | - | - | - | 1 (25.0) | - | 0.505 |
| Indeterminate | 5 (17.9) | 1 (16.7) | 3 (25.0) | - | - | - | - | - | - | 1 (33.3) | 0.470 |
| Alternative | 18 (64.3) | 3 (50.0) | 7 (58.3) | - | 3 (100.0) | - | - | - | 3 (75.0) | 2 (66.7) | 0.468 |
| **Autoimmune phenomena^f^** |  |  |  |  |  |  |  |  |  |  |  |
| Arthralgia | 75 (75.0) | 25 (80.6) | 17 (65.4) | 11 (100.0) | 9 (90.0) | 4 (66.7) | - | 1 (100.0) | 7 (70.0) | 1 (25.0) | 0.069 |
| Arthritis | 27 (27.0) | 11 (35.5) | - | 11 (100.0) | 2 (20.0) | 1 (16.7) | - | - | 2 (20.0) | - | <0.001 |
| Raynaud’s phenomenon | 44 (44.0) | 12 (38.7) | 15 (57.7) | 3 (27.3) | 8 (80.0) | 1 (16.7) | - | - | 3 (30.0) | 2 (50.0) | 0.193 |
| Sicca complaints | 51 (51.0) | 13 (41.9) | 21 (80.8) | 4 (36.4) | 4 (40.0) | 2 (33.3) | - | - | 7 (70.0) | - | 0.050 |
| Mechanic’s hands | 11 (11.0) | 6 (19.4) | 1 (3.8) | 2 (18.2) | 2 (20.0) | 1 (16.7) | - | - | 1 (10.0) | - | 0.469 |
| Myalgia | 55 (55.0) | 24 (77.4) | 13 (50.0) | 4 (36,4) | 4 (40.0) | 3 (50.0) | - | - | 7 (70.0) | - | 0.054 |
| Muscle weakness | 36 (36.0) | 13 (41.9) | 8 (30.8) | 3 (27,3) | 5 (50.0) | 3 (50.0) | - | - | 4 (40.0) | - | 0.667 |
| Data are expressed as mean and standard deviation or numbers and percentage within the diagnosis group.  CTD-ILD = connective tissue disease related interstitial lung disease; ASS: antisynthetase syndrome; RA-ILD; rheumatoid arthritis associated interstitial lung disease; Ssc; systemic sclerosis; PM/DM; polymyositis/dermatomyositis; IBM; inclusion body myositis; SLE; systemic lupus erythematosus.  ^a^ Other CTD-ILD: IgG4 related disease (n = 3), Bechterew’s disease (n = 1)  FVC = forced vital capacity; FEV1 = forced expiratory volume in 1 second; DLCO = diffusing capacity of the lung for carbon monoxide; CA 15-3 = cancer antigen 15-3; CCL18 = CC chemokine ligand 18; CC16 = Clara cell secretory protein; SP-D = surfactant protein D; YKL-40 = chitinase-3-like protein 1; HRCT = high resolution computed tomography; UIP = usual interstitial pneumonia  ^b^ n = 97, data expressed mean and standard deviation in percentage of predicted  **^c^** n = 99, data expressed as mean and standard deviation in kU/l (CA 15-3) or ng/ml (CC16, CCL18, SP-D, YKL-40)  **^d^** n = 97, data expressed as numbers and percentage  **^e^** n = 28, data expressed as numbers and percentage  **^f^** n = 99 data expressed as numbers and percentage. Data on autoimmune phenomena of IBM (n=1) is missing  **^f^** p < 0.05, differences between the CTD-ILD subgroups are calculated by a one way ANOVA for continuous variables (post-hoc Bonferroni test) or Chi-Square test/ Fisher’s Exact Test for dichotomous variables | | | | | | | | | | | |

| **Supplementary table 2 Baseline characteristics of 294 ILD patients without established CTD (non-CTD-ILD)** | | | | | | | | |
| --- | --- | --- | --- | --- | --- | --- | --- | --- |
| **Subjects** |  |  |  |  |  |  |  |  |
|  | **All Non-CTD-ILD** | **IPF** | **Unclassifiable IIP** | **HP** | **NSIP** | **COP** | **Other ILD^a^** | **P^f^** |
| N | 294 | 68 | 106 | 53 | 31 | 11 | 25 |  |
| Age | 65.1 (10.7) | 67.4 (9.9) | 66.3 (10.1) | 64.0 (8.5) | 62.0 (9.6) | 67.0 (14.4) | 59.2 (15.4) | 0.006 |
| Sex (m), % | 196 (66.7) | 55 (80.9) | 69 (65.1) | 34 (64.2) | 17 (54.8) | 4 (36.4) | 17 (68.0) | 0.022 |
| History of smoking, % | 212 (72.4) | 58 (85.3) | 76 (71.1) | 35 (66.0) | 17 (54.8) | 4 (36.4) | 22 (88.0) | 0.001 |
| **Pulmonary function test^b^** |  |  |  |  |  |  |  |  |
| FVC | 81.2 (19.0) | 80.1 (15.6) | 78.7 (19.3) | 80.7 (20.0) | 84.2 (23.3) | 97.0 (17.7) | 85.3 (15.4) | 0.042 |
| FEV1 | 82.3 (19.8) | 84.1 (15.6) | 81.0 (18.9) | 78.8 (22.6) | 83.4 (27.9) | 90.6 (19.1) | 85.3 (16.5) | 0.392 |
| DLCO | 46.2 (16.5) | 41.6 (13.4) | 45.0 (15.9) | 46.6 (19.3) | 44.6 (11.1) | 70.3 (14.0) | 52.8 (16.2) | <0.001 |
| **Pneumoprotein^c^** | |  |  |  |  |  |  |  |
| CA 15-3 | 69.8 (68.9) | 78.3 (68.8) | 66.2 (40.7) | 95.1 (120.2) | 57.2 (40.5) | 27.3 (13.6) | 41.7 (24.3) | 0.005 |
| CC16 | 21.2 (15.1) | 25.3 (14.8) | 22.0 (15.1) | 22.6 (17.9) | 15.8 (11.4) | 9.8 (5.3) | 13.9 (10.5) | 0.001 |
| CCL18 | 177.3 (118.3) | 162.1 (69.1) | 165.0 (81.2) | 194.4 (175.2) | 230.4 (180.2) | 110.3 (67.5) | 209.0 (141.6) | 0.033 |
| SP-D | 115.1 (200.8) | 107.0 (123.5) | 94.0 (141.8) | 208.9 (373.3) | 125.0 (166.3) | 24.7 (28.3) | 63.0 (94.5) | 0.010 |
| YKL-40 | 151.2 (137.8) | 164.8 (169.1) | 157.3 (140.1) | 122.1 (78.5) | 121.9 (105.9) | 140.9 (71.3) | 183.8 (169.0) | 0.350 |
| **HRCT scan^d^** |  |  |  |  |  |  |  |  |
| UIP | 85 (29.4) | 56 (82.4) | 19 (17.9) | 9 (18.0) | - | - | 1 (4.2) | <0.001 |
| Probable UIP | 40 (13.8) | 6 (8.8) | 28 (26.4) | 2 (4.0) | 2 (6.7) | - | 2 (8.3) | <0.001 |
| Indeterminate | 51 (17.6) | 6 (8.8) | 21 (19.8) | 8 (16.0) | 8 (26.7) | 2 (18.2) | 6 (25.0) | 0.217 |
| Alternative | 113 (39.1) | - | 38 (35.8) | 31 (62.0) | 20 (66.7) | 9 (81.8) | 15 (62.5) | <0.001 |
| **Histopathology^e^** | |  |  |  |  |  |  |  |
| UIP | 7 (7.9) | 15 (100) | 8 (25.8) | 1 (4.8) | - | - | - | <0.001 |
| Probable UIP | 15 (16.9) | - | 6 (19.4) | 1 (4.8) | - | - | - | 0.062 |
| Indeterminate | 43 (48.3) | - | 4 (12.9) | 4 (19.0) | 4 (44.4) | 1 (16.7) | 2 (28.6) | 0.067 |
| Alternative | 294 | - | 13 (41.9) | 15 (71.4) | 5 (55.6) | 5 (83.3) | 5 (71.4) | <0.001 |
| Data are expressed as mean and standard deviation or numbers and percentage within the diagnosis group.  ILD = interstitial lung disease; non-CTD-ILD = ILD without established CTD; IPF = idiopathic pulmonary fibrosis; HP = hypersensitivity pneumonitis; Unclassifiable IIP = unclassifiable idiopathic interstitial pneumonia; NSIP = non-specific interstitial pneumonia; COP = cryptogenic organizing pneumonia; FVC = forced vital capacity; FEV1 = forced expiratory volume in 1 second; DLCO = diffusing capacity of the lung for carbon monoxide; CA 15-3 = cancer antigen 15-3; CCL18 = CC chemokine ligand 18; CC16 = Clara cell secretory protein; SP-D = surfactant protein D; YKL-40 = chitinase-3-like protein 1; HRCT = high resolution computed tomography; UIP = usual interstitial pneumonia  ^a^ Other ILD: desquamative interstitial pneumonia (n = 5), drug induced (n = 5), interstitial pneumonia with autoimmune features (n = 6), pneumoconiosis (n = 4), respiratory bronchiolitis interstitial pneumonia (n = 3) , sarcoidosis (n = 2).  ^b^ n = 282, data expressed mean and standard deviation in percentage of predicted  **^c^** n = 275, data expressed as mean and standard deviation in kU/l (CA 15-3) or ng/ml (CC16, CCL18, SP-D, YKL-40)  **^d^** n = 289, data expressed as numbers and percentage  **^e^** n = 89, data expressed as numbers and percentage  **^f^** p < 0.05, differences between other ILD subgroups are calculated by a one way ANOVA for continuous variables (post-hoc Bonferroni test) or Chi-Square test/Fisher’s Exact Test for dichotomous variables | | | | | | | | |

| **Supplementary table 3** | | | **Frequency of myositis antibodies in CTD-ILD patients** | | | | | | | |  |  |
| --- | --- | --- | --- | --- | --- | --- | --- | --- | --- | --- | --- | --- |
| **Antibody** | **N (%)** | |  | | | | | | | |  |  |
|  | **CTD-ILD** | **ASS** | | **Sjogren’s syndrome** | **RA-ILD** | **Ssc** | **PM/DM** | **IBM** | **SLE** | **Mixed CTD-ILD** | **Other CTD-ILD** ^a^ | **P^f^** |
| N | 100 | 31 | | 26 | 11 | 10 | 6 | 1 | 1 | 10 | 4 |  |
| **MSA** |  |  | |  |  |  |  |  |  |  |  |  |
| EJ | 5 (5.0) | 1 (3.2) | | 1 (3.8) | - | - | 1 (16.7) | - | - | 1 (10.0) | - | 0.233 |
| Jo-1 | 27 (27.0) | 21 (67.7) | | - | 2 (18.2) | - | 1 (16.7) | - | - | 3 (30.0) | - | <0.001 |
| MDA5 | 2 (2.0) | - | | - | - | - | 2 (33.3) | - | - | - | - | 0.577 |
| Mi-2α | 1 (1.0) | - | | - | - | - | - | - | - | 1 (10.0) | - | 0.789 |
| Mi-2β | 7 (7.0) | 1 (3.2) | | 1 (3.8) | 3 (2,3) | - | 1 (16.7) | - | - | - | 1 (25.0) | 0.224 |
| NXP2 | 4 (4.0) | - | | 1 (3.8) | - | - | - | 1 (100.0) | 1 (100.0) | 1 (10.0) | - | 0.183 |
| OJ | 1 (1.0) | - | | - | - | - | - | - | - | 1 (10.0) | - | 0.789 |
| PL-12 | 11 (11.0) | 3 (9.7) | | 6 (23.1) | - | 1 (10.0) | - | - | - | 1 (10.0) | - | 0.386 |
| PL-7 | 5 (5.0) | 3 (9.7) | | 2 (7.7) | - | - | - | - | - | - | - | 0.660 |
| SAE1 | 2 (2.0) | - | | - | - | - | - | - | - | - | - | 0.194 |
| SRP | 2 (2.0) | - | | - | - | - | - | - | - | 2 (20.0) | - | 0.135 |
| TIF1-γ | 1 (1.0) | - | | 1 (3.8) | - | - | - | - | - | - | - | 0.823 |
| **MAA** |  |  | |  |  |  |  |  |  |  |  |  |
| Ku | 1 (1.0) | - | | - | 1 (9.1) | - | - | - | - | - | - | 0.969 |
| PM/Scl 100 | 13 (13.0) | 1 (3.2) | | - | 2 (18.2) | 4 (40.0) | 1 (16.7) | - | 1 (100.0) | 3 (30.0) | 1 (25.0) | 0.004 |
| PM/Scl 75 | 13 (13.0) | 2 (6.5) | | - | - | 6 (60.0) | 1 (16.7) | - | - | 3 (30.0) | 1 (25.0) | 0.023 |
| Ro52 | 67 (67.0) | 26 (83.9) | | 24 (92.3) | 5 (45.5) | 4 (40.0) | 3 (50.0) | - | - | 4 (40.0) | 1 (25.0) | 0.004 |
| Data are expressed as numbers and percentage of positive antibodies within each ILD diagnosis group. Weak positive antibodies are excluded.  CTD = connective tissue disease; ILD = interstitial lung disease; ASS: antisynthetase syndrome; RA-ILD; rheumatoid arthritis associated interstitial lung disease; Ssc; systemic sclerosis; PM/DM; polymyositis/dermatomyositis; IBM; inclusion body myositis; SLE; systemic lupus erythematosus; MSA = myositis specific antibodies; MAA = myositis associated antibodies  ^a^ Other CTD-ILD: IgG4 related disease (n = 3), Bechterew’s disease (n = 1)  ^b^ p < 0.05, difference between CTD-ILD patients calculated by Chi-Square test/ Fisher’s Exact Test. | | | | | | | | | | | | |

| **Supplementary table 4** | | **Frequency of myositis antibodies in patients with non-CTD-ILD** | | | | | | | |
| --- | --- | --- | --- | --- | --- | --- | --- | --- | --- |
| **Antibody** | **N (%)** |  |  |  |  |  |  |  |  |
|  | **All non-CTD-ILD** | **IPF** | **Unclassifiable IIP** | **HP** | **NSIP** | **COP** | **Other ILD**^a^ |  | **P**^b^ |
|  |  |  |  |  |  |  |  |  |  |
| N | 294 | 68 | 106 | 53 | 31 | 11 | 25 |  |  |
| **MSA** |  |  |  |  |  |  |  |  |  |
| EJ | 5 (1.7) | 4 (5.9) | 1 (0.9) | - | - | - | - |  | 0.089 |
| Jo-1 | 16 (5.4) | 6 (8.8) | 3 (2.8) | 4 (7.5) | - | 2 (18.2) | 1 (4.0) |  | 0.277 |
| MDA5 | 6 (2.0) | - | 4 (3.8) | 1 (1.9) | 1 (3.2) | - | - |  | 0.251 |
| Mi-2α | 5 (1.7) | 1 (1.5) | 4 (3.8) | - | - | - | - |  | 0.138 |
| Mi-2β | 61 (20.7) | 18 (26.5) | 19 (17.9) | 14 (26.4) | 3 (9.7) | 4 (36.4) | - |  | 0.012 |
| NXP2 | 3 (1.0) | 1 (1.5) | 1 (0.9) | 1 (1.9) | - | - | - |  | 0.604 |
| OJ | 6 (2.0) | 1 (1.5) | 1 (0.9) | 2 (3.8) | - | 1 (9.1) | 1 (4.0) |  | 0.525 |
| PL-12 | 10 (3.4) | 1 (1.5) | 2 (1.9) | 3 (5.7) | 3 (9.7) | - | 1 (4.0) |  | 0.450 |
| PL-7 | 19 (6.5) | 5 (7.4) | 5 (4.7) | 6 (11.3) | 3 (9.7) | - | - |  | 0.177 |
| SAE1 | 14 (4.8) | 8 (11.8) | 3 (2.8) | 3 (5.7) | - | - | - |  | 0.039 |
| SRP | 27 (9.2) | 8 (11.8) | 9 (8.5) | 1 (1.9) | 4 (12.9) | 1 (9.1) | 4 (16.0) |  | 0.304 |
| TIF1-γ | 23 (7.8) | 1 (1.5) | 15 (14.2) | 4 (7.5) | 1 (3.2) | 2 (18.2) | - |  | 0.039 |
| **MAA** |  |  |  |  |  |  |  |  |  |
| Ku | 19 (5.4) | 6 (8.8) | 8 (7.5) | - | 2 (6.5) | - | 3 (4.0) |  | 0.116 |
| PM/Scl 100 | 20 (6.5) | 5 (7.4) | 8 (7.5) | 6 (11.3) | - | - | 1 (4.0) |  | 0.139 |
| PM/Scl 75 | 33 (11.2) | 7 (10.3) | 9 (8.2) | 7 (13.2) | 3 (9.7) | 1 (9.1) | 6 (24.0) |  | 0.531 |
| Ro52 | 75 (25.5) | 10 (14.7) | 31 (29.2) | 8 (15.1) | 17 (54.8) | 3 (27.3) | 6 (24.0) |  | <0.001 |
| Data are expressed as numbers and percentage of positive antibodies within each ILD diagnosis group. Weak positive antibodies are excluded.  CTD = connective tissue disease; ILD = interstitial lung disease; non-CTD-ILD = ILD without established CTD; IPF = idiopathic pulmonary fibrosis; HP = hypersensitivity pneumonitis; Unclassifiable IIP = unclassifiable idiopathic interstitial pneumonia; NSIP = non-specific interstitial pneumonia; COP = cryptogenic organizing pneumonia; MSA = myositis specific antibodies; MAA = myositis associated antibodies  ^a^ Other ILD: desquamative interstitial pneumonia (n = 5), drug induced (n = 5), interstitial pneumonia with autoimmune features (n = 6), pneumoconiosis (n = 4), respiratory bronchiolitis interstitial pneumonia (n = 3) , sarcoidosis (n = 2).  ^b^ p < 0.05, differences between other ILD patients calculated by Chi-Square test/ Fisher’s Exact Test. | | | | | | | | | |

| **Supplementary table 5** | | **Associations of myositis antibodies with CTD-ILD and IPF patients** | | | | | | | |
| --- | --- | --- | --- | --- | --- | --- | --- | --- | --- |
| **Antibody** | **CTD-ILD (n = 100)** | | | **IPF (n = 68)** | | | | | |
|  | Number Neg | Number Weak pos | Number Pos | Number Neg | Number Weak pos | Number Pos | OR p  OR wp**^a^** | 95% CI^b^ | p^c^ |
| EJ | 95 | - | 5 | 64 | - | 4 | 0.84 | 0.22-3.26 | 0.803 |
|  |  |  |  |  |  |  |  |  |  |
| Jo-1 | 72 | 1 | 27 | 60 | 2 | 6 | 3.75 | 1.45-9.68 | 0.006 |
|  |  |  |  |  |  |  | 0.42 | 0.04-4.71 | 0.479 |
| Ku | 98 | 1 | 1 | 58 | 4 | 6 | 0.10 | 0.01-0.84 | 0.034 |
|  |  |  |  |  |  |  | 0.15 | 0.02-1.36 | 0.091 |
| MDA5 | 97 | 1 | 2 | 63 | 5 | - | - |  |  |
|  |  |  |  |  |  |  | 0.13 | 0.02-1.14 | 0.065 |
| Mi-2α | 99 | - | 1 | 65 | 2 | 1 | 0.66 | 0.04-10.68 | 0.768 |
|  |  |  |  |  |  |  | - |  |  |
| Mi-2β | 91 | 2 | 7 | 45 | 5 | 18 | 0.19 | 0.08-0.49 | 0.001 |
|  |  |  |  |  |  |  | 0.20 | 0.04-1.06 | 0.058 |
| NXP2 | 95 | 1 | 4 | 64 | 3 | 1 | 2.70 | 0.29-24.67 | 0.380 |
|  |  |  |  |  |  |  | 0.23 | 0.02-2.21 | 0.200 |
| OJ | 99 | - | 1 | 65 | 2 | 1 | 0.66 | 0.04-10.7 | 0.768 |
|  |  |  |  |  |  |  | - |  |  |
| PL-12 | 89 | - | 11 | 66 | 1 | 1 | 8.16 | 1.03-64.76 | 0.047 |
|  |  |  |  |  |  |  | - |  |  |
| PL-7 | 95 | - | 5 | 63 | - | 5 | 0.66 | 0.18-2.39 | 0.529 |
|  |  |  |  |  |  |  | - |  |  |
| PM/Scl 100 | 87 | - | 13 | 62 | 1 | 5 | 1.85 | 0.63-5.47 | 0.264 |
|  |  |  |  |  |  |  | - |  |  |
| PM/Scl 75 | 86 | 1 | 13 | 59 | 2 | 7 | 1.27 | 0.48-3.38 | 0.627 |
|  |  |  |  |  |  |  | 0.39 | 0.03-3.87 | 0.387 |
| Ro52 | 31 | 2 | 67 | 55 | 3 | 10 | 11.89 | 5.36-26.38 | <0.001 |
|  |  |  |  |  |  |  | 1.18 | 0.19-7.47 | 0.858 |
| SAE1 | 98 | 2 | - | 58 | 2 | 8 | - |  |  |
|  |  |  |  |  |  |  | 0.59 | 0.08-4.32 | 0.605 |
| SRP | 90 | 8 | 2 | 48 | 12 | 8 | 0.13 | 0.03-0.65 | 0.013 |
|  |  |  |  |  |  |  | 0.36 | 0.14-0.93 | 0.035 |
| TIF1-γ | 96 | 3 | 1 | 65 | 2 | 1 | 0.68 | 0.04-11.0 | 0.784 |
|  |  |  |  |  |  |  | 1.02 | 0.17-6.25 | 0.987 |
| CTD = connective tissue disease; IPF = idiopathic pulmonary fibrosis  **^a^** OR: odds ratio for positive level (OR p); odds ratio for weakly positive level (OR wp).  ^b^ 95% confidence interval of odds ratio’s  ^c^ Logistic regression analysis of CTD-ILD versus IPF patients with positive, weakly positive and negative antibody, with predicted probability for CTD-ILD. | | | | | | | | | |
